# Causal Estimands for Aging-Relevant Outcomes in the Presence of Death as a Competing or Truncating Event

**DOI:** 10.1093/geroni/igaf122.1857

**Published:** 2025-12-31

**Authors:** L Paloma Rojas-Saunero, Yixuan Zhou, Elizabeth Rose Mayeda

**Affiliations:** University of California, Los Angeles, Los Angeles, California, United States; University of California, Los Angeles, Los Angeles, California, United States; University of California, Los Angeles, Los Angeles, California, United States

## Abstract

Research aiming to identify causal mechanisms and intervention targets to prevent dementia and other aging-related outcomes often faces challenges when death acts as a competing or truncating event. For example, some studies have reported inverse associations between cigarette smoking and dementia risk, which could be attributable to “survival bias.” Substantial progress has been made in developing statistical estimators to address this issue, but less attention has been given to aligning research questions and appropriate estimators, an essential step for meaningful interpretation of results. This work evaluates potential causal contrasts (i.e., estimands) relevant to incident outcomes, such as dementia diagnosis, as well as longitudinal outcomes, such as cognitive change, in the presence of death as a competing or truncating event. We compare these estimands, discuss potential sources of selection bias, and clarify the interpretation of estimates through simulations grounded in population-based cohort studies. Our findings aim to guide researchers in selecting estimators that align with their research questions, ultimately improving the validity of inferences in aging research.

